# Glucocorticoid/Adiponectin Axis Mediates Full Activation of Cold-Induced Beige Fat Thermogenesis

**DOI:** 10.3390/biom11111573

**Published:** 2021-10-23

**Authors:** Liping Luo, Lu Wang, Yan Luo, Estevan Romero, Xin Yang, Meilian Liu

**Affiliations:** 1 Department of Biochemistry and Molecular Biology, University of New Mexico Health Sciences Center, Albuquerque, NM 87131, USA; llp3253@163.com (L.L.); luwang112@163.com (L.W.); luoyan_2018@163.com (Y.L.); estevan22@unm.edu (E.R.); xinyang@salud.unm.edu (X.Y.); 2 Department of Psychiatry, The Second Xiangya Hospital, Central South University, Changsha 410011, China; 3 Department of Endocrinology and Metabolism, Metabolic Syndrome Research Center, The Second Xiangya Hospital, Central South University, Changsha 410011, China; 4 Key Laboratory of Diabetes Immunology, National Clinical Research Center for Metabolic Diseases, The Second Xiangya Hospital, Central South University, Changsha 410011, China; 5 Autophagy, Inflammation and Metabolism Center, University of New Mexico Health Sciences Center, Albuquerque, NM 87131, USA

**Keywords:** glucocorticoid, adiponectin, WAT, beige adipocytes, PPARγ

## Abstract

Glucocorticoids (GCs), a class of corticosteroids produced by the adrenal cortex in response to stress, exert obesity-promoting effects. Although adaptive thermogenesis has been considered an effective approach to counteract obesity, whether GCs play a role in regulating cold stress-induced thermogenesis remains incompletely understood. Here, we show that the circulating levels of stress hormone corticosterone (GC in rodents) were significantly elevated, whereas the levels of adiponectin, an adipokine that was linked to cold-induced adaptive thermogenesis, were decreased 48 h post cold exposure. The administration of a glucocorticoid hydrocortisone downregulated adiponectin protein and mRNA levels in both WAT and white adipocytes, and upregulated thermogenic gene expression in inguinal fat. In contrast, mifepristone, a glucocorticoid receptor antagonist, enhanced adiponectin expression and suppressed energy expenditure in vivo. Mechanistically, hydrocortisone suppressed adiponectin expression by antagonizing PPARγ in differentiated 3T3-L1 adipocytes. Ultimately, adiponectin deficiency restored mifepristone-decreased oxygen consumption and suppressed the expression of thermogenic genes in inguinal fat. Taken together, our study reveals that the GCs/adiponectin axis is a key regulator of beige fat thermogenesis in response to acute cold stress.

## 1. Introduction

Brown and beige adipocytes, referred to as lipid-burning cells, dissipate energy in the form of heat, and are widely recognized as a potential therapeutic approach for the treatment of obesity and its associated disorders. The sympathetic nervous system (SNS) richly supports brown adipose tissue (BAT) and is also arborized in white adipose tissue (WAT) at a certain level [1,2]. Upon cold exposure, SNS releases norepinephrine in adipose tissue, thereby, activating β3 adrenoceptor signaling and thermogenic program in adipocytes [1,2]. 

The neuroendocrine hormones, including the thyroid hormones triiodothyronine (T3) and thyroxine (T4), glucocorticoids (GCs), and the adrenal medullary hormones catecholamine and dopamine, play a pivotal role in metabolic homeostasis maintenance [3,4,5] However, the mechanisms underlying neuroendocrine regulation of thermogenic program and cold adaptation remain largely unknown.

GCs are a class of corticosteroids that are critical for the regulation of metabolic, cardiovascular, immunologic, and homeostatic functions in response to various types of stress in humans and in rodents [6,7]. Although it has been well considered that GCs mobilize glucose, amino acids, and fatty acids, whether GCs are involved in cold-induced thermogenesis remains to be established. Cortisol in humans and corticosterone in rodents are the principal circulating GCs and are secreted under the control of the hypothalamic–pituitary–adrenal (HPA) axis [8]. In mice, fecal corticosterone excretion and plasma adrenocorticotropic hormone (ACTH) increased after 24-hour cold exposure [9]. 

The correlation between cold stress and the cortisol circulating level has also been assessed in humans, and the effects of cold exposure on serum cortisol levels are contradictory [10,11,12,13,14,15,16]. Some studies showed that serum levels of cortisol and/or ACTH were elevated by cold stress [10,11,12,13,14,15]. However, other studies reported a decrease or no effect on the cortisol levels in response to cold exposure [14,16,17]. Therefore, more studies are in need to address the controversy about the correlation between GCs and cold stress.

Stress hormone GCs demonstrate obesogenic and pro-diabetic properties. The increased level of glucocorticoid explains the metabolic abnormalities, such as obesity, hypertension, hyperlipidemia, and glucose intolerance in Cushing’s syndrome with pituitary adenoma [6,18,19]. Chronic GC excess has been also considered to induce obesity at least in part through suppressing BAT activation and browning of WAT in rodents and in humans [20,21,22,23,24,25]. The complexity of GC physiology has been appreciated in the past several years. 

Luijten et al. demonstrated that GC administration for 2 weeks lowered UCP1 expression only at thermoneutrality conditions, and the obesity-promoting effects of GC do not require UCP1 under both room temperature and thermoneutrality [26]. Moreover, GC acutely increased BAT activity in humans, implying the primary involvement of GC in cold-induced thermogenesis [27]. In the same study, GC action in human brown adipocytes is distinct from that in rodent cells where cortisol substantially suppressed isoprenaline-stimulated respiration and UCP-1 [27]. While cold-induced fecal corticosterone excretion was reported [9], whether circulating GC senses short-term cold stress and modulates thermogenesis at such condition is largely unknown.

GCs bind to the glucocorticoid receptor (GR) to form a complex and substantially facilitate the translocation of its own receptor into the cell nucleus and transcriptional control of target genes involved in inflammation, gluconeogenesis, and adipocyte differentiation [28,29]. Upon activation, dimerized GR binds to GC response elements (GREs) and activates gene transcription (Bamberger et al., 1996, Schacke and Rehwinkel, 2004). On the other hand, the Glucocorticoid–GR system has been shown to mediate the promoting effects of chronic stress on adipose tissue inflammation and abnormal glucose and lipid metabolism in obese rats [30]. 

Constitutive deletion of GR in adipocytes attenuated diet- and aging-induced adiposity accompanied with impaired WAT lipolysis and reduced thermogenesis, whereas had a little effect on BAT UCP1 in short-term cold exposed mice [31]. One recent study showed that conditional adipocyte-specific GR invalidation improved glucose and lipid profiles with an expansion of fat mass [32]. Interestingly, Glantschnig et al. using UCP1-dependent, BAT-specific GR knockout mice demonstrated a negligible role of GR in brown adipocytes in BAT function and systemic metabolism [33]. These studies implied that glucocorticoid-GR signaling plays an unrevealed role in WAT metabolism. However, whether glucocorticoid-GR signaling is required for acute GCs modulation of WAT browning and energy expenditure remains to be established. 

In the current study, we observed that circulating levels of corticosterone are elevated, while adiponectin levels are decreased by acute cold stress. Moreover, hydrocortisone administration induces thermogenic gene expression in WAT and suppresses adiponectin expression and secretion in vivo and in differentiated adipocytes. In contrast, treatment with the GR antagonist mifepristone suppressed thermogenesis and cold-induced energy expenditure, an effect that is diminished in adiponectin deficient mice. Mechanistically, hydrocortisone downregulates adiponectin expression via a PPARγ-dependent mechanism in adipocytes. Our study reveals that elevated glucocorticoid in circulation mediates acute cold stress-induced full activation of beige fat thermogenesis by suppressing adiponectin expression and secretion.

## 2. Materials and Methods

### 2.1. Materials

Antibodies against PGC1α were purchased from Millipore. Antibodies against UCP1 and C/EBPβ were from Abcam. β-Actin antibody was purchased from Cell Signaling, and monoclonal anti-β-Tubulin antibody was bought from Sigma Aldrich. Anti-mouse IgG (H+L) with HRP conjugated was from Promega. Adiponectin antibody was kindly provided by Dr. Feng Liu’s laboratory at the University of Texas Health at San Antonio. Hydrocortisone, mifepristone, PPARγ agonist rosiglitazone, and PPARγ antagonist GW9662 were purchased from Sigma (St. Louis, MO, United States).

### 2.2. Mice

Adiponectin knockout mice were kindly provided by Dr. Philipp Scherer at the University of Texas Southwestern Medical Center and transferred from Dr. Feng Liu’s laboratory at the University of Texas Health at San Antonio. C57BL/6J wild type mice were purchased from Jackson Laboratory. All the animals were housed in a pathogen-free barrier facility with a 12-h light/12-h dark cycle with free access to food and water. All animal experimental protocols were reviewed and approved by the Animal Care Committee of the University of New Mexico Health Sciences Center, which is registered with the USDA (ID: 85-R-0014), certified by Office of Laboratory Animal Welfare (ID: A3350-01) and accredited by AAALAC (ID: 000222). For cold stress studies, the mice were housed in an environmental chamber at 30 °C followed with 48 h of either 22 °C or 6 °C and then euthanized for western blot analysis, as described in our previous study [34,35].

### 2.3. Hydrocortisone or Mifepristone Administrations

For hydrocortisone intraperitoneal administration, hydrocortisone was diluted by PBS. We administered 10-week-old C57BL/6J male mice with hydrocortisone or PBS through intraperitoneal injection at 80 mg/kg body weight every 12 h for four times. A calorimetry study was performed 48 h before the first injection at 22 °C. At 48 h after the first administration, the mice were euthanized for western blot analysis. For mifepristone intraperitoneal administration, mifepristone was dissolved in dimethyl sulfoxide (DMSO) and diluted by vehicle solution, which contained 5% Tween 80 and 5% PEG 400 in PBS. We administered 10-week-old adiponectin WT and KO male mice with mifepristone or vehicle solution through intraperitoneal injection at 5 mg/kg body weight once a day for two times. A calorimetry study was performed 48 h before the first administration at 22 °C. At 4 h after first administration, the mice were housed in an environmental chamber at 6 °C for another 44 h and then euthanized for western blot analysis.

### 2.4. Calorimetry Study

The 10-week-old male mice were individually housed in eight separate Promethion Metabolic Phenotyping Systems (Sable Systems International, Las Vegas, NV, United States) coupled with a temperature controllable chamber. Oxygen consumption (VO_2_), carbon dioxide release (VCO_2_), food intake, water intake, and the activity of each animal were monitored at room temperature (22 °C) and in cold stress conditions (6 °C) for the indicated time. The data were analyzed using the ExpeData software associated with the system. Oxygen consumption was normalized by body weight as we described previously [36,37]. 

### 2.5. Cell Culture and Treatment

3T3-L1 fibroblast cells (preadipocytes) were obtained from ATCC, Ltd., (Manassas, VA, United States), and brown preadipocytes were provided by Dr. Jiandie Lin (University of Michigan, Ann Arbor, MI, United States). 3T3-L1 preadipocytes or brown preadipocytes were cultured and differentiated into mature adipocytes as described in our previous studies [38,39]. The cells were changed into a fresh culture medium for 4 h and then treated with hydrocortisone, mifepristone, rosiglitazone or GW9662 at the indicated concentrations for 24 h. Cells and culture medium were harvested for western blot analysis or qPCR.

### 2.6. Western Blot

Tissue samples and cell lysates were prepared in ice-cold lysis buffer, and the general procedures were used for western blot as described in our previous study [38]. For the tissue sample preparation, frozen BAT samples were cut, and approximately 1 mg tissue samples were homogenized for western blot analysis. For the inguinal WAT (iWAT), tissue samples around the lymph node in the inguinal fat were used for the western blot considering that UCP1^+^ cells are predominantly in the inguinal region rather than the dorsolumbar region after a 2-day cold exposure within a posterior subcutaneous fat pad, a key feature of adipocyte heterogeneity [40,41]. For all western blot data presented in this study, the x axis indicates the individual mouse or the individual cell treatment.

### 2.7. Statistics

The effects of various treatments on the expression levels of genes in tissue and cells were analyzed by a Student’s *t* test for comparisons of two groups or ANOVA for comparisons of more than two groups. The statistical analysis of energy expenditure in vivo was performed by ANOVA. The individual in vitro experiment was repeated for at least three times. Data are presented as the means ± S.E.M unless otherwise specified. *p* < 0.05 was considered as significant.

## 3. Results 

### 3.1. Circulating Levels of Corticosterone Are Elevated by Acute Cold Stress

Circulating levels of adiponectin were suppressed by cold exposure [42,43], and this provides a mechanism underlying cold-induced adipose-resident group 2 innate lymphoid cells (ILC2) activation and thermogenesis [35]. We asked whether stress-responded neuroendocrine hormones are altered by cold stress. 

**Figure 1:**
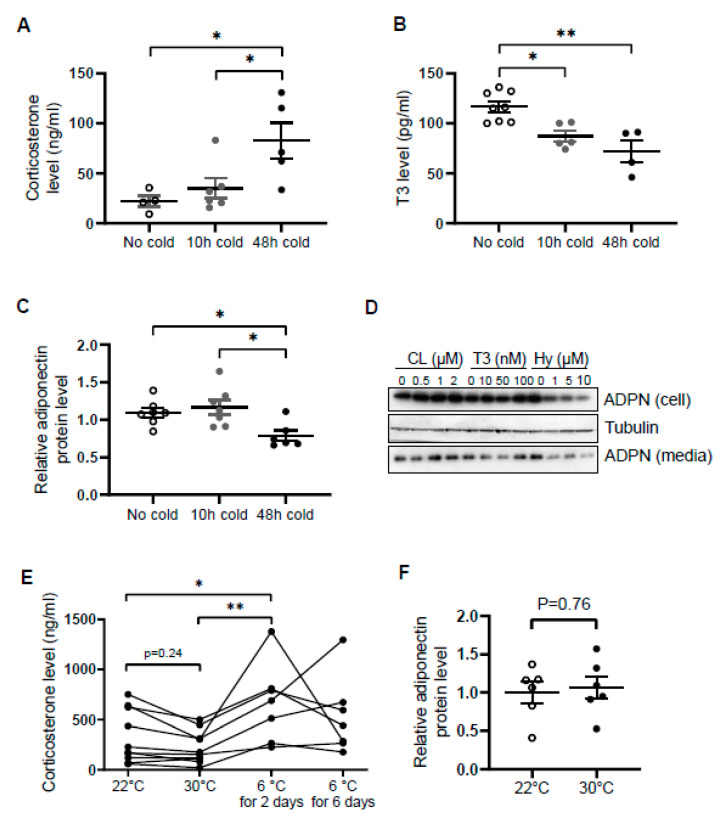
Circulating levels of corticosterone are elevated while adiponectin levels are suppressed by acute cold stress. Acute cold stress increased the circulating levels of corticosterone (**A**), while suppressed the levels of T3 (**B**) and adiponectin (**C**) in serum. White dots, No cold group (mice housed at 22 °C, *n* = 4–8); gray dots, 10 h cold group (mice housed at 6 °C for 10 h, *n* = 5–7); black dots, 48 h cold group (mice housed at 6 °C for 48 h, *n* = 4–6). (**D**). Treatment of hydrocortisone suppressed expression and release of adiponectin in differentiated 3T3-L1 adipocytes in a dose-dependent manner despite no significant effect when treated with CL or T3 for 24 h. (**E**) Circulating levels of corticosterone did not significantly alter at 30 °C or after 6 days of cold exposure. Each black dot indicates the individual mouse. (**F**) Adiponectin expression showed little change between mice housed at 22 °C and 30 °C. White dots, mice housed at 22 °C, *n* = 6; gray dots, mice housed at 30 °C, *n* = 6. ADPN, adiponectin. The data in Figure 1A–C are presented as mean ± S.E.M. * *p* < 0.05, ** *p* < 0.01.

Similar to norepinephrine levels in WAT [34], circulating levels of corticosterone were increased by cold exposure for 48 h in male mice (Figure 1A), while the levels of T3 and adiponectin in serum were decreased by acute cold stress (Figure 1B,C). To investigate whether the downregulation of circulating adiponectin was resulted from the altered neuroendocrine hormones, we treated differentiated 3T3-L1 adipocytes with β3 adrenoceptor agonist CL316,243, T3 and hydrocortisone with different doses for 24 h. We found that the treatment of hydrocortisone but not CL or T3 downregulated both expression levels and secretion levels of adiponectin in adipocytes (Figure 1D), suggesting that acute cold stress downregulates adiponectin expression through GC. 

In agreement with this, the inducing effects of cold stress on GC were diminished during the extension of cold stress (Figure 1E), suggesting an acute elevation of corticosterone by cold stress. In addition, the mild stress from 30 °C to 22 °C had no significant effect on the levels of corticosterone and adiponectin (Figure 1E,F).

### 3.2. Hydrocortisone Administration Enhanced Oxygen Consumption and Thermogenic Gene Expression

**Figure 2:**
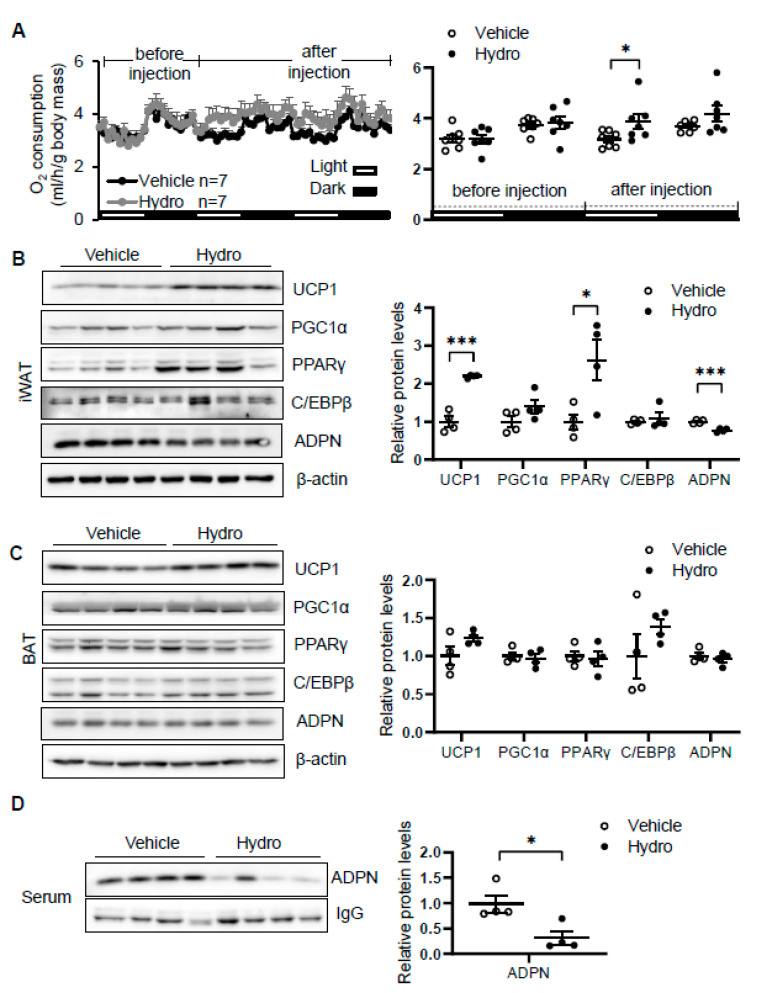
Hydrocortisone increases energy expenditure and suppresses adiponectin expression in adipose tissue. (**A**) Administration of hydrocortisone increased oxygen (O_2_) consumption of wild type (WT) mice. The average O_2_ consumption was normalized to whole-body mass. Hydrocortisone treatment upregulated the expression of thermogenesis genes *Ucp1* and *Pparγ* and downregulated the protein levels of adiponectin in iWAT (**B**) but not in BAT (**C**). (**D**) Hydrocortisone treatment decreased the levels of adiponectin in serum. White dots, vehicle group (*n* = 4); black dots, hydrocortisone group (*n* = 4). Hydro, hydrocortisone. The statistical data are presented as the mean ± S.E.M. * *p* < 0.05, *** *p* < 0.001.

Elevation of circulating corticosterone by cold stress promoted us to investigate whether corticosterone plays a role in regulating thermogenesis. We administered vehicle or 80 mg/kg hydrocortisone twice a day through intraperitoneal injection for 2 days in 2-month-old male C57/BL6J mice. The results showed that hydrocortisone administration increased oxygen consumption with little effect on food intake, animal activity, and respiratory quotient (RQ) in vivo (Figure 2A and Figure S1A–C). In addition, the expression levels of thermogenic genes *Ucp1* and *PPARγ* in iWAT (Figure 2B) but not in BAT (Figure 2C) were induced by hydrocortisone administration, indicating that corticosterone promotes cold-induced thermogenesis. Consistent with our recent finding that adiponectin inhibits thermogenesis in adipose tissue [35], hydrocortisone administration suppresses adiponectin expression in iWAT (Figure 2B) and in circulation (Figure 2D). These results suggest that cold-glucocorticoid pathway may induce thermogenesis by suppressing adiponectin.

### 3.3. Blocking Glucocorticoid Receptor Signaling Leads to Decreased Energy Expenditure and Thermogenesis in an Adiponectin-Dependent Manner 

**Figure 3:**
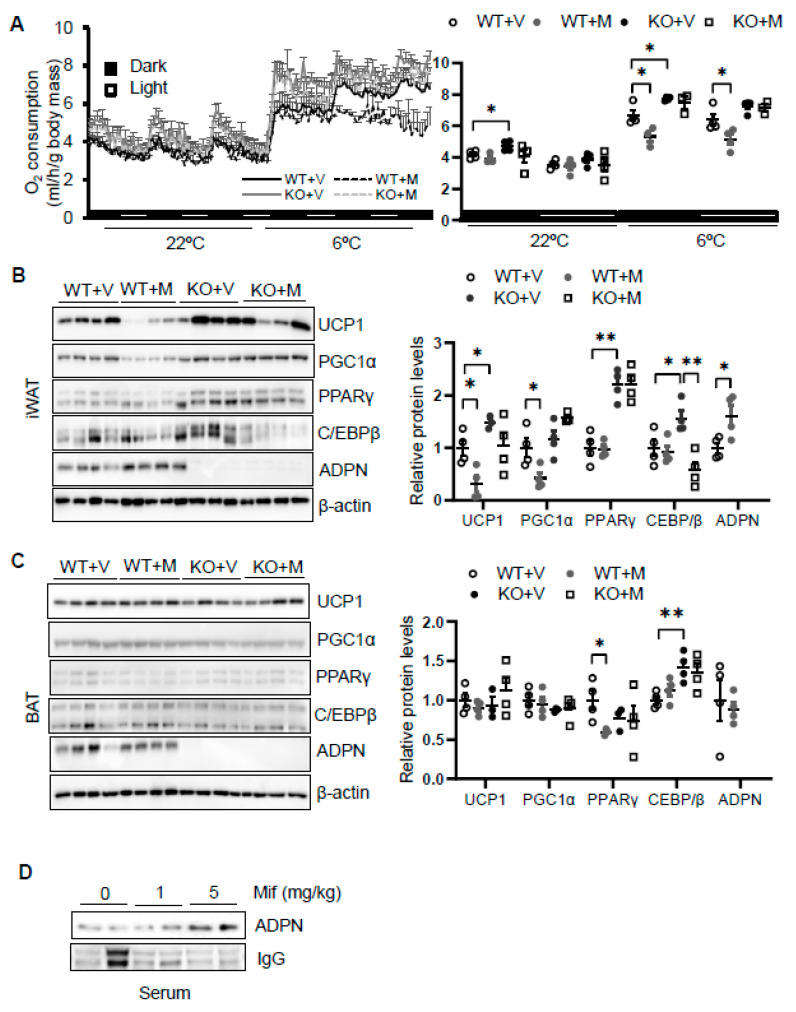
Blocking glucocorticoid receptor signaling inhibits cold-induced energy expenditure through upregulating adiponectin. (**A**) Administration of mifepristone suppressed acute cold-induced O_2_ consumption throughout the light and dark cycle. The average O_2_ consumption was normalized to whole-body mass. WT + V, wild type mice treated with vehicle; WT + M, wild type mice treated with mifepristone; KO+V, adiponectin KO mice treated with vehicle; KO+M, adiponectin KO mice treated with mifepristone. Under cold conditions, the administration of mifepristone downregulated the expression of thermogenesis genes *Ucp1* and *Pgc1α*, and these effects were diminished in iWAT (**B**) but not in BAT (**C**) of adiponectin KO mice. White dots, WT mice with vehicle injection (*n* = 4); gray dots, WT mice with mifepristone injection (*n* = 5); black dots, adiponectin KO mice with vehicle injection (*n* = 4); and white square, adiponectin KO mice with mifepristone injection (*n* = 4). (**D**) Treatment of mifepristone increased circulating levels of adiponectin. The data in Figure 3A–C are presented as the mean ± S.E.M. * *p* < 0.05, ** *p* < 0.01.

To dissect the role of glucocorticoid in regulating thermogenesis, we administered mifepristone 5 mg/kg intraperitoneally in 2-month-old male wild type. As a result, administration of mifepristone suppressed cold (6 °C for 48 h)-induced energy expenditure with no effect at room temperature with no significant effects on food intake, animal activities, and respiratory quotient (Figure 3A and Figure S1D–F). Consistent with this, mifepristone treatment downregulated the expression levels of thermogenic genes *Ucp1* and *Pgc1α* in iWAT (Figure 3B) but not in BAT (Figure 2C) under cold condition, supporting the promoting effect of glucocorticoid on thermogenesis. On the other hand, mifepristone treatment induced adiponectin expression in iWAT and in circulation (Figure 3B,D). Furthermore, the suppressing effects of mifepristone on thermogenic gene expression and energy expenditure were abolished in adiponectin deficient mice (Figure 3A,B), suggesting that adiponectin downregulation mediates glucocorticoid-promoted thermogenesis. 

### 3.4. Hydrocortisone Suppressed Adiponectin Transcription via GR-Dependent Mechanism in Adipocytes

**Figure 4:**
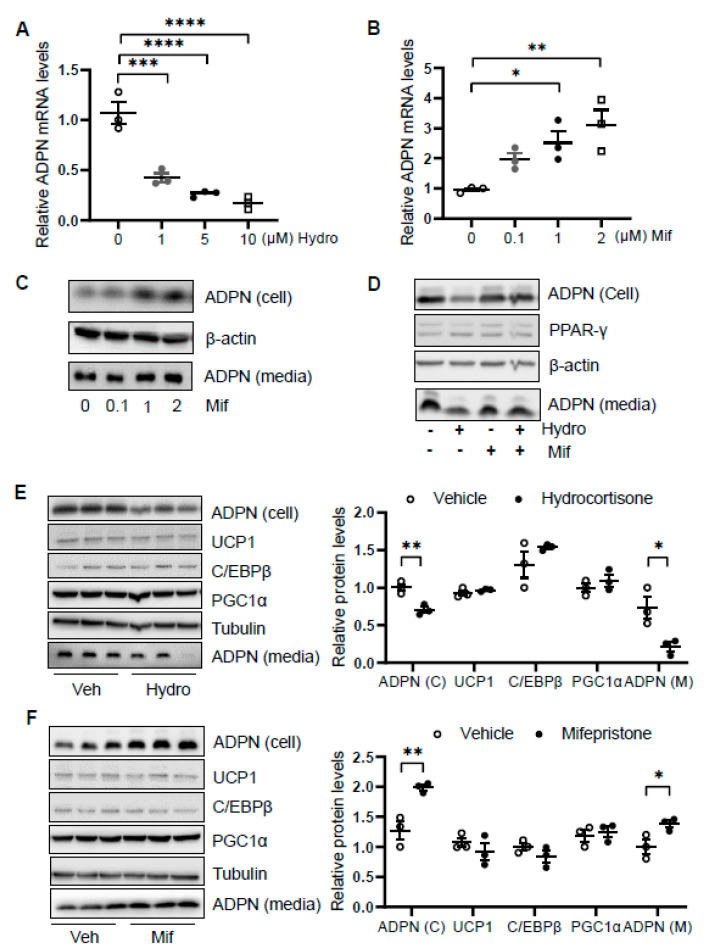
Glucocorticoid receptor signaling transcriptionally suppresses adiponectin in adipocytes. Differentiated 3T3-L1 adipocytes or brown adipocytes were treated with hydrocortisone or mifepristone for 24 h at indicated concentrations. For the co-treatment, 3T3-L1 adipocytes were pre-treated by 2 μM mifepristone for 1 h followed by the co-treatment of 2 μM hydrocortisone. (**A**) Hydrocortisone treatment suppressed adiponectin mRNA levels in differentiated 3T3-L1 adipocytes in a dose-dependent manner. White dots, vehicle group (*n* = 3); gray dots, 1 μM hydrocortisone group (*n* = 3); black dots, 5 μM hydrocortisone group (*n* = 3); and white square, 10 μM hydrocortisone group (*n* = 3). Treatment with GC receptor antagonist mifepristone upregulated adiponectin in both mRNA level (**B**) and protein expression/secretion (**C**) in a dose-dependent manner in differentiated 3T3-L1 adipocytes. White dots, vehicle group (*n* = 3); gray dots, 0.1 μM mifepristone group (*n* = 3); black dots, 1 μM mifepristone group (*n* = 3); and white square, 2 μM mifepristone group (*n* = 3). (**D**) The suppressing effect of hydrocortisone on adiponectin expression and secretion was restored by mifepristone treatment in 3T3-L1 adipocytes. (**E**) Hydrocortisone treatment suppressed expression and secretion of adiponectin but had no significant effects on the expression of thermogenesis genes in the differentiated brown adipocytes. White dots, vehicle group (*n* = 3); black dots, hydrocortisone group (*n* = 3). (**F**) Mifepristone treatment induced the expression and secretion of adiponectin without significant effects on the expression of thermogenesis genes in the differentiated brown adipocytes. White dots, vehicle group (*n* = 3); black dots, mifepristone group (*n* = 3). Veh, vehicle. Hydro, hydrocortisone. Mif, mifepristone. The data in Figure 4A,B,E,F are presented as the mean ± S.E.M. * *p* < 0.05, ** *p* < 0.01, *** *p* < 0.001, **** *p* < 0.0001.

We then asked how glucocorticoid suppresses adiponectin expression. We treated differentiated 3T3-L1 adipocytes with or without hydrocortisone at different doses for 24 h and found that hydrocortisone treatment downregulated adiponectin mRNA and protein levels (Figure 4A and Figure 1D). In contrast, mifepristone treatment upregulated the levels of adiponectin in mRNA and protein as well as the secretion of adiponectin in differentiated 3T3-L1 adipocytes (Figure 4B,C). Along with this, blocking GR with mifepristone rescued the suppressing effect of hydrocortisone on adiponectin expression and secretion (Figure 4D). 

In addition, we treated differentiated brown adipocytes and observed that although hydrocortisone treatment suppressed adiponectin expression and secretion, the expression levels of thermogenic genes *Ucp1*, *C/ebpβ,* and *Pgc1α* were little affected in brown adipocytes (Figure 4E). Moreover, mifepristone treatment upregulated adiponectin expression and secretion with little effects on thermogenic gene expression in brown adipocytes (Figure 4F), suggesting that the inducing effect of hydrocortisone on thermogenic gene expression is not mediated by an intrinsic pathway in adipocytes.

### 3.5. Glucocorticoid Suppresses Adiponectin Expression by Regulating PPARγ Activity

**Figure 5:**
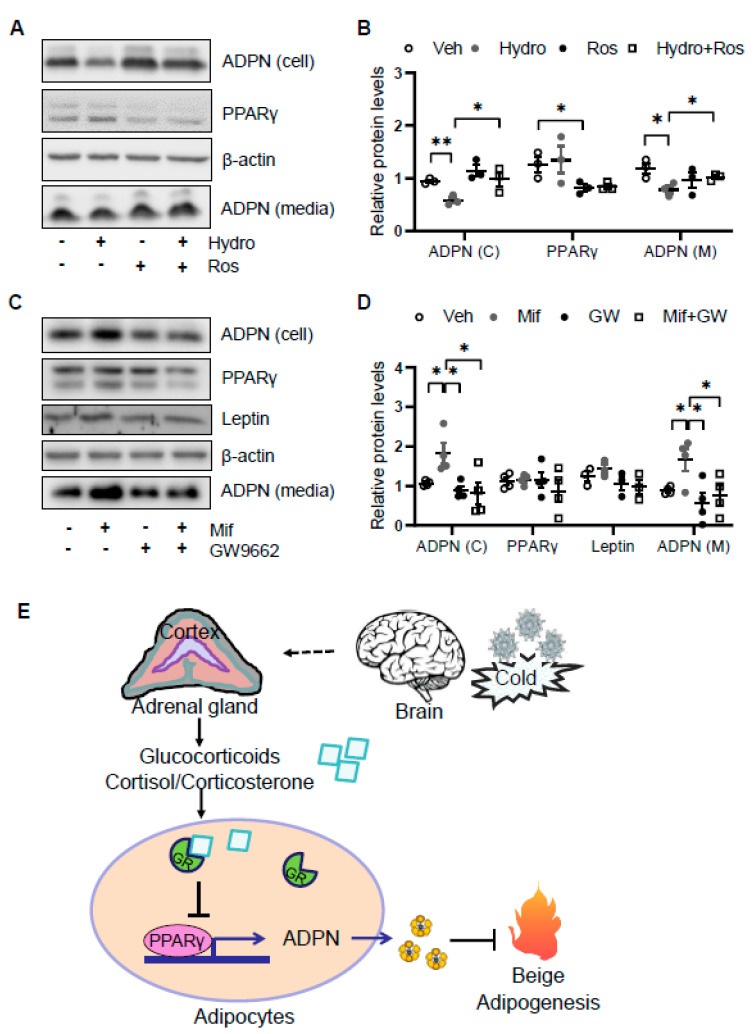
Glucocorticoid receptor signaling suppresses adiponectin expression by inactivating PPARγ. (**A**) The suppressing effects of GCs on adiponectin expression was attenuated by PPARγ agonist rosiglitazone in differentiated 3T3-L1 adipocytes. Cells were treated with 2 μM rosiglitazone for 1 h followed by the co-treatment of 2 μM hydrocortisone for 24 h. (**B**) The statistical analysis of the results in Figure 5A. White dots, vehicle group (*n* = 3); gray dots, hydrocortisone group (*n* = 3); black dots, rosiglitazone group (*n* = 3); and white square, hydrocortisone and rosiglitazone cotreatment group (*n* = 3). (**C**) The inducing effects of mifepristone on adiponectin were suppressed by the treatment of PPARγ antagonist GW9662 in differentiated 3T3-L1 adipocytes. Cells were treated with 20 μM PPARγ antagonist GW9662 for 1 h followed with the co-treatment of 2 μM mifepristone for 24 h. (**D**) The statistical analysis of the results in Figure 5C. White dots, vehicle group (*n* = 4); gray dots, mifepristone group (*n* = 4); black dots, GW9662 group (*n* = 4); and white square, mifepristone and GW9662 cotreatment group (*n* = 4). Hydro, hydrocortisone; Ros, rosiglitazone; Mif, Mifepristone; and GW, GW9662. (**E**) A schematic model showing that cold stress-elevated GCs promote thermogenesis by suppressing adiponectin production. The data in Figure 5B,D are presented as the mean ± S.E.M. * *p* < 0.05, ** *p* < 0.01.

As the glucocorticoid receptor has been shown to physically interact with PPARγ [7], we investigated if PPARγ is involved in glucocorticoid suppressed adiponectin expression in adipocytes. We found that the suppressing effects of hydrocortisone on adiponectin expression and secretion were reversed by the cotreatment with PPARγ agonist rosiglitazone (Figure 5A,B). Consistent with this, treatment of PPARγ antagonist GW9662 abrogated the inducing effect of mifepristone on adiponectin expression and secretion (Figure 5C,D). These results suggest that glucocorticoid suppresses adiponectin expression in a PPARγ-dependent manner in adipocytes (Figure 5E).

## 4. Discussion

SNS plays a predominant role in cold-induced thermogenesis and energy expenditure by producing and releasing norepinephrine. In response to cold stress, HPA axis is also activated and drives the production and release of GCs from the adrenal gland [44,45,46]. However, it is incompletely understood whether and how GCs are involved in cold-induced adaptive thermogenesis. Our present study shows that corticosterone in circulation is elevated by acute cold stress and that its acute excess enhances oxygen consumption and thermogenic gene expression in beige fat through suppression of adiponectin in mice (Figure 5E). In addition, acute treatment of GC suppresses adiponectin expression via GR and PPARγ-dependent mechanisms in adipocytes (Figure 5E). Our study reveals that GCs play a critical role in adiponectin biosynthesis and cold adaptation.

In response to stressors, such as exercise and imminent danger, the activated HPA axis promotes the production of adrenal cortex hormone GCs and adrenal medullary hormones, including norepinephrine (noradrenaline), epinephrine (adrenaline), and dopamine. These adrenal gland hormones are then released into blood and target a variety of tissue/organs to regulate the specific metabolic pathways. Adrenaline in circulation has been shown to be elevated in response to cold stress, and the administration of adrenaline significantly increases the metabolic rate in vivo [46,47]. 

Along with this, lacking epinephrine by ablation of phenylethanolamine N-methyl transferase, the enzyme that catalyzes the conversion of norepinephrine to epinephrine, impairs cold-induction of thermogenic genes *Ucp1* and *Pgc1α* [48]. However, the involvement of GCs in cold-induced thermogenesis and energy expenditure and the correlation between circulation levels of GCs and cold stress remain controversial [10,11,12,13,14,15,16,17]. 

Our study suggests that GC production is stimulated by acute cold stress and contributes to cold-induced adaptive thermogenesis in adipose tissue. Upon hydrocortisone treatment, oxygen consumption as well as thermogenic gene expression in beige adipose tissue is induced. In contrast, suppressing GR signaling using GR antagonist reduces cold-induced energy expenditure and thermogenic gene expression in adipose tissue. These results suggest an important role of GCs in acute cold stress-induced adaptive thermogenesis. We were aware that beige fat only contributes to a small portion of thermogenesis. Additional mechanisms, such as the central action of GCs/adiponectin axis, may be involved in GC-enhanced energy metabolism.

The existing factors, including the high basal innervation of BAT and unique signature of inguinal WAT versus BAT, may explain the reduced sensitivity of BAT to cold stress. Adipose-resident immune cells may contribute to the activation of beige fat in response to cold but lesser extent in BAT. Type 2 inflammatory pathways, including ILC2, regulatory T cells (Treg), M2 macrophages, eosinophils, γδT cells, and their own derived cytokines, play a vital role in regulating beige adipocyte development and activation despite no significant effects on BAT function [49,50,51,52,53,54]. 

Along this line, adiponectin acts as a molecular brake on cold-induced type 2 immune response and thermogenesis in beige fat by regulating the activation of ILC2 with less significant effects on BAT [35]. In addition, UCP1-independent mechanisms also play a role in cold-induced energy burning in beige fat. Ca(2+)-ATPase 2b (SERCA2b)-mediated Ca^2+^ cycling is required for ATP-dependent thermogenesis as a result of enhanced glycolysis, tricarboxylic acid metabolism, and pyruvate dehydrogenase activity in beige adipocytes, which was found more active in beige fat rather than BAT [55]. 

Interestingly, adiponectin has been shown to control intracellular Ca^2+^ flux [56,57], although whether adiponectin plays a role in regulating Ca^2+^ cycling is unclear. On the other hand, PEPCK appears to be suppressed and mediates the effect of glucocorticoids in BAT but not in WAT [58]. Therefore, type 2 immune response, PEPCK pathway, SERCA2b-driven Ca^2+^ cycling, and less innervation in beige fat may contribute to the selective induction by GC on beige fat thermogenesis. Further clarification is needed.

It is also possible that BAT-specific PPARγ-interacting factors counteract the effect of GR. Glantschnig et al. demonstrated that GR plays a negligible role in thermogenesis and metabolism in BAT by using BAT-specific GR knockout mice [33]. PPARγ likely selectively binds to various target genes in different fat depots through the interaction with its co-regulators, such as Early B cell factor-2 (Ebf2) and Transducin-like enhancer protein 3 (TLE3) [59,60,61]. By facilitating PPARγ binding to BAT-selective genes, Ebf2 drives the activation of brown fat-specific genes [60] while TLE3 acts as a white-selective PPARγ cofactor to promote lipid storage and counter the BAT program [61]. However, little is known about whether GC plays a role in regulating the Ebf2/PPARγ pathway that counteracts the effect of glucocorticoids in BAT. 

Adiponectin exerts anti-diabetic effects [62,63,64,65], and its expression and circulating levels are downregulated by obesity in human subjects [66,67]. However, the role of adiponectin in the regulation of energy homeostasis and thermogenesis remains controversial. Several studies showed that adiponectin promotes energy expenditure and cold-induced browning effect through its central and peripheral actions [42,68,69,70,71]. However, other studies have suggested that adiponectin may be a negative regulator of energy expenditure and thermogenesis [72,73,74,75,76,77]. 

Using the same mouse model with dietarily challenged mice [65], we recently found that adiponectin plays an inhibitory role in regulating cold-induced adipose-resident ILC2s, type 2 immunity, and energy expenditure [35]. To validate if adiponectin exerts anti-thermogenic effects, we characterized another adiponectin KO mouse strain from the Jackson Laboratory after backcrossing this strain with C57BL/6 for four generations [35]. The results also indicated that adiponectin acts as a negative regulator of thermogenesis in adipose tissue [35]. In support of this, our present study shows that GCs enhance energy expenditure by suppressing adiponectin expression.

The accumulated evidence suggests that GCs and adiponectin play opposite roles in the maintenance of metabolic homeostasis. GCs activate catabolic processes and induce insulin resistance, while adiponectin acts primarily as an insulin sensitizer [19]. Despite a pro-inflammatory effect of adiponectin under certain conditions, both GC and adiponectin have been well considered as anti-inflammatory hormones [78,79,80,81,82,83,84,85,86,87]. 

Therefore, it has gained increased attention whether GCs control metabolic pathways and inflammatory response through regulating adiponectin. The effects of GCs on adiponectin expression have been explored in cell, animal and clinical study settings, while no consensus has been reached [88]. Some studies show that GC treatment suppresses adiponectin expression in mRNA and/or protein in adipocytes and decreases the levels of adiponectin in serum and adipose tissue [89,90,91,92,93,94,95,96]. 

Whereas other studies suggest no effect of GCs on adiponectin or even inducing effect of GCs in vitro and in animals [97,98,99,100,101]. The controversy may have resulted from the diversity in the dose, cell line, drug, and/or fat depot [88]. Our study demonstrates that acute treatment of GCs downregulates circulating levels and expression of adiponectin in subcutaneous WAT but not in brown adipose tissue. In addition, GC treatment suppresses adiponectin transcription via inactivation of PPARγ. In agreement with this, GR physically interacts with PPARγ to suppress the immune response by targeting TNF-α and IL-1β [102]. 

Glucocorticoids (GCs) are a class of corticosteroids that exert an immunosuppressive property and are remarkably efficacious in managing various acute disease manifestations of inflammatory and autoimmune disorders [103,104]. In addition to the reduction of inflammation, corticosteroids also suppress overactive immune system responses and help with hormonal imbalances [103]. The anti-inflammatory response has been linked to the browning of white fat and substantial improved energy expenditure [49,50,51,54]. 

In alignment with this, we observed that short-term administration of hydrocortisone induces beige fat thermogenesis and increases energy expenditure, indicating the potential metabolic benefits of GC acute treatment. Whereas, the long-term action of GCs may be distinct given their obesogenic and pro-diabetic effects. In support of this, patients who consume corticosteroids have been suggested to be closely monitored for glucose intolerance and hyperlipidemia [104,105]. There is also an increased risk of acute vascular events, including myocardial infarction, shortly after starting high-dose steroids [104,105,106,107,108].

In addition to glucocorticoid receptor, hydrocortisone and mifepristone have been shown to bind to other nuclear receptors, and such additional signaling pathways may also contribute to the phenotype we observed. The mineralocorticoid receptor (MR) is a hormone-activated transcription factor that binds with hydrocortisone to inhibit browning via a cell autonomous mechanism and to blunt cold-induced UCP1 expression in BAT [109,110,111]. In contrast, overexpression of MR in adipocytes increases fat mass and insulin resistance [112], whereas there was no obvious metabolic phenotype in adipocyte-specific MR knock out mice [113]. It is possible that both GR and MR may mediate the increased energy consumption by hydrocortisone.

On the other hand, mifepristone acts as a progesterone receptor antagonist, and progesterone treatment showed induction of UCP1 expression in brown adipocytes [114]. Although the progesterone receptor is expressed in white adipocytes [115], there is no evidence showing a role of progesterone treatment or progesterone receptor in regulating WAT browning. The effects of mifepristone on thermogenesis are likely mediated mainly via GR-dependent mechanism. 

In summary, we found that a new pathway involving GCs/adiponectin played an important role in acute cold-induced thermogenesis. Cold stress-elevated GCs suppressed adiponectin expression in adipose tissue and enhanced a thermogenic program. Our study demonstrated that stress hormone GCs turn on a thermogenic program by downregulating adiponectin production.

## Supplementary Materials

The following are available online at https://www.mdpi.com/article/10.3390/biom11111573/s1. Figure S1: The Supplementary results showed that the administration of hydrocortisone or mifepristone had no significant effects on food intake, animal activities, and respiratory quotient.
