# Supplementary figures and images for: Glucocorticoid/Adiponectin Axis Mediates Full Activation of Cold-Induced Beige Fat Thermogenesis
Source: Biomolecules. 2021 Oct 23;11(11):1573. doi: 10.3390/biom11111573 (PMC8615797; doi:10.3390/biom11111573)

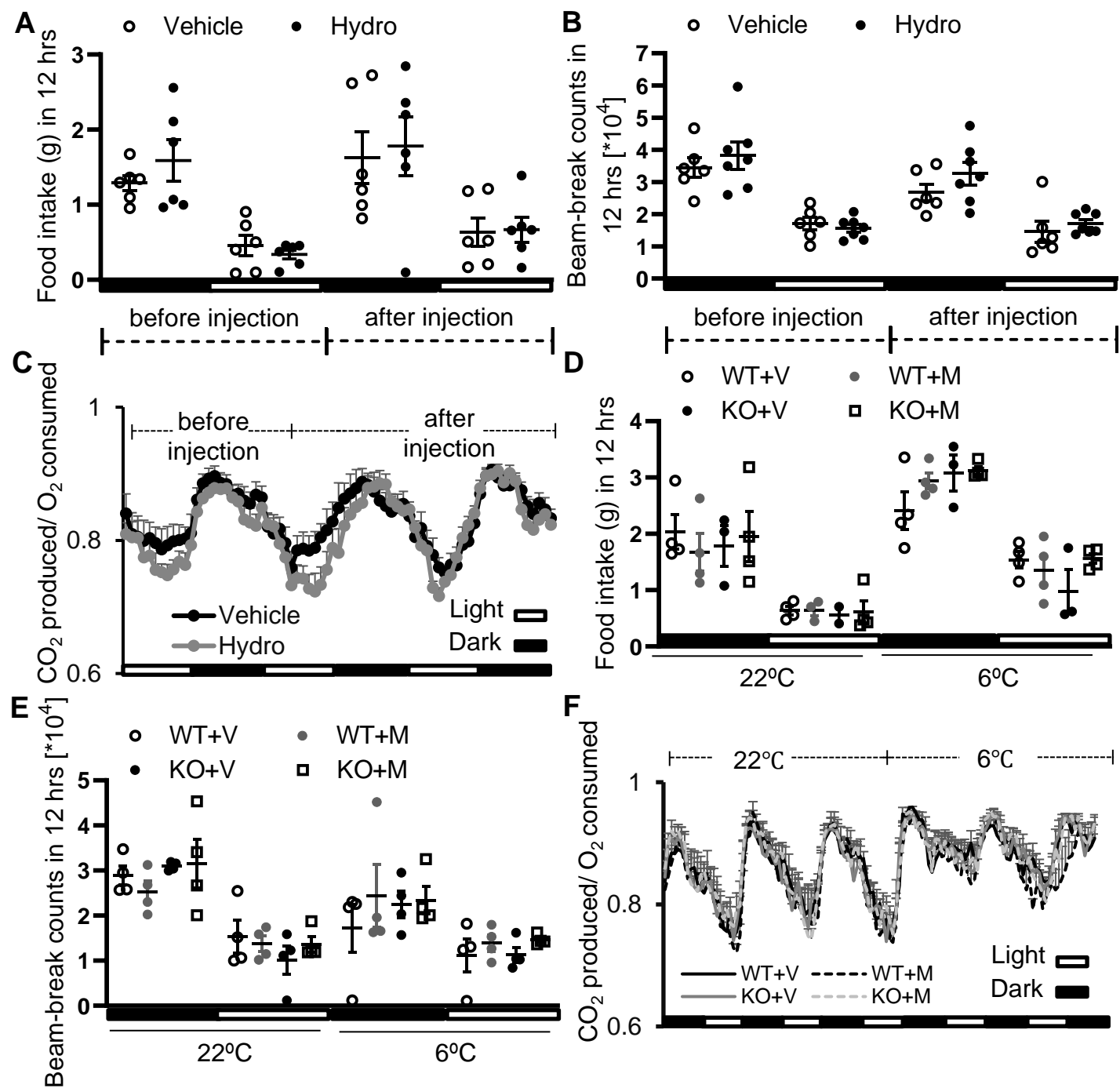

Fig.S1

Supplement: Supplementary file 1 [file biomolecules-11-01573-s001.zip › biomolecules-1410713-supplementary.pdf]
